# Higher Risk of HEV Transmission and Exposure among Blood Donors in Europe and Asia in Comparison to North America: A Meta-Analysis

**DOI:** 10.3390/pathogens12030425

**Published:** 2023-03-08

**Authors:** Annika Wolski, Sven Pischke, Ann-Kathrin Ozga, Marylyn M. Addo, Thomas Horvatits

**Affiliations:** 1 Department of Medicine, University Medical Center Hamburg-Eppendorf, 20251 Hamburg, Germany; 2 German Center for Infection Research (DZIF), Hamburg-Lübeck-Borstel-Riems and Heidelberg Partner Sites, 20251 Hamburg, Germany; 3 Institute of Medical Biometry and Epidemiology, University Medical Center Hamburg-Eppendorf, 20251 Hamburg, Germany; 4 Institute for Infection Research and Vaccine Development (IIRVD), University Medical Center Hamburg-Eppendorf, 20251 Hamburg, Germany; 5 Gastromedics Health Center, 7000 Eisenstadt, Austria

**Keywords:** hepatitis E virus, HEV, meta-analysis, blood donors

## Abstract

Background and aims: The increasing number of diagnosed hepatitis E virus (HEV) infections in Europe has led to the implementation of the testing of blood products in various countries. Many nations have not yet implemented such screening. To assess the need for HEV screening in blood products worldwide, we conducted a systematic review and meta-analysis assessing HEV RNA positivity and anti-HEV seroprevalence in blood donors. Methods: Studies reporting anti-HEV IgG/IgM or HEV RNA positivity rates among blood donors worldwide were identified via predefined search terms in PubMed and Scopus. Estimates were calculated by pooling study data with multivariable linear mixed-effects metaregression analysis. Results: A total of 157 (14%) of 1144 studies were included in the final analysis. The estimated HEV PCR positivity rate ranged from 0.01 to 0.14% worldwide, with strikingly higher rates in Asia (0.14%) and Europe (0.10%) in comparison to North America (0.01%). In line with this, anti-HEV IgG seroprevalence in North America (13%) was lower than that in Europe (19%). Conclusions: Our data demonstrate large regional differences regarding the risk of HEV exposure and blood-borne HEV transmission. Considering the cost–benefit ratio, this supports blood product screening in high endemic areas, such as Europe and Asia, in contrast to low endemic regions, such as the U.S.

## 1. Introduction

Globally, hepatitis E is the most common cause of enterically transmitted hepatitis [1]. Anti-HEV IgG seroprevalence rates of 18% up to 30% were reported in the Netherlands and Germany in 2013, and even higher rates of over 50% were reported in some areas of France [2]. In humans, genotypes 1–4 are of the highest relevance. Genotypes 1 and 2 exclusively infect humans and are endemic in tropical areas, such as Asia, the south of Africa, and parts of Central America. As a source of fecal–oral transmission, contaminated drinking water can lead to local outbreaks with sometimes fulminant courses [3]. By contrast, HEV genotype 3 is the predominant genotype in Europe and Australia as well as North and South America. Genotype 3 is transmitted zoonotically. Consumption of raw pork seems to be the most relevant risk factor in genotype 3 regions [4]. Genotype 4 is also transmitted through pork meat but occurs primarily in Asia and plays virtually no role in Europe [5]. The majority of HEV infections are asymptomatic and self-limiting. Only a small proportion of patients develop elevated transaminases and hepatic dysfunction. In the case of HEV genotype 3 infections, older men and patients with preexisting liver disease are considered to be particularly at risk for a severe course [6]. Persistent HEV infections (chronic hepatitis E) have been found in various immunocompromised patient populations. These include solid organ transplant patients as well as stem cell transplant patients, HIV-infected patients, chemotherapy-receiving patients, and patients with chronic inflammatory diseases who are permanently receiving immunosuppressive therapy [7,8]. Patients suffering from chronic hepatitis E are at risk of developing life-threatening cirrhosis over the next five years [9]. Up to 50% of organ transplant recipients who acquire an HEV infection develop chronic hepatitis E, putting them at high risk for developing cirrhosis [6]. In addition to foodborne transmission, HEV can be transmitted parenterally, via transfusion of blood products [10,11,12,13]. Immunosuppressed patients receiving blood transfusions are particularly at risk. In the UK, the Netherlands, Japan, Austria, Germany, and France, general testing of blood products for HEV has been introduced in recent years. Despite the fact that recorded HEV infections are on the rise in many countries and the risk of chronification of hepatitis E in immunocompromised patients is high, most countries worldwide do not routinely test blood products for HEV. These countries are still evaluating the situation and need valid data depicting the risk of blood-borne HEV infections in their country in comparison to that in other nations.

To evaluate the risk of HEV-positive blood products as well as the risk of HEV exposition in blood donors, a systematic review and meta-analysis was performed comparing the rate of HEV RNA positivity and anti-HEV seroprevalence in blood donors worldwide. As HEV genotype 3 infections are endemic in both North America and Europe, it is adequate to focus on the comparison of PCR and serology positivity rates on these two continents; by contrast, several genotypes are endemic in Asia (genotypes 1, 3, and 4) and Africa (genotypes 1, 2, and 3), and therefore, an inhomogeneous picture exists for these continents. Even if different HEV genotype 3 subtypes are present in North America and Europe, the focus on these two continents allows a relevant comparison.

## 2. Methods

### 2.1. Search Strategy and Selection Criteria

The literature search was conducted in two different types of databases: PubMed and Scopus. In both databases, the literature search was performed by using the terms “Hepatitis e” or “HEV” in combination with the terms “blood donors” or “transfusion” or “blood donation” or “blood testing”. A total of 1144 articles were identified and screened for duplicates and reviews, and all duplicates and reviews were removed. Published articles were thoroughly reviewed for possible inclusion. This analysis is reported in line with the Preferred Reporting Items for Systematic Reviews and Meta-Analyses (PRISMA).

### 2.2. Inclusion and Exclusion Criteria

The inclusion criteria were as follows: identification of the test used (ELISA or PCR) and confirmation of use according to the manufacturer’s instructions; inclusion of only specified blood donors (e.g., studies reporting on healthy individuals or volunteers were excluded). Only articles written in English and study cohorts with more than 50 blood donors were included in the final analysis. Studies that did not meet these study quality criteria were excluded from further analysis.

### 2.3. Data Extraction

Data were stratified by author, journal, year of publication, continent, country, diagnostic assay, number of blood donors, anti-HEV IgG and IgM seroprevalence, and HEV-RNA positivity in different data sets. Additionally, we categorized blood donors as female or male. If different diagnostic assays were used in one study or if different study cohorts were divided according to gender, a study could contain several data sets.

### 2.4. Study Quality

The identified articles were assessed for study quality according to a set scheme. Data were assessed on the basis of their methodological quality according to the Joanna Briggs Institute’s well-established critical appraisal tool for prevalence studies. Studies were assessed by A.W. and discussed with T.H. accordingly. Any disagreements were resolved by discussing with a third investigator (S.P.).

### 2.5. Statistical Analysis

The HEV RNA positivity rate was estimated by pooling the study data separately for each country and continent with a linear mixed effects regression analysis using restricted maximum likelihood. We included the test, year of publication, and methodological quality score as further independent variables. If possible, interaction terms, e.g., for the publication year and test, were also included. Heterogeneity was checked via the quantity *I*^2^, and publication bias was conducted via a funnel plot. Odds ratios along with 95% confidence intervals are given. The analysis was checked using R (version 3.6.1) and the *metafor* package. For the rainforest plots, the R package *metaviz* was used.

## 3. Results

**Figure 1:**
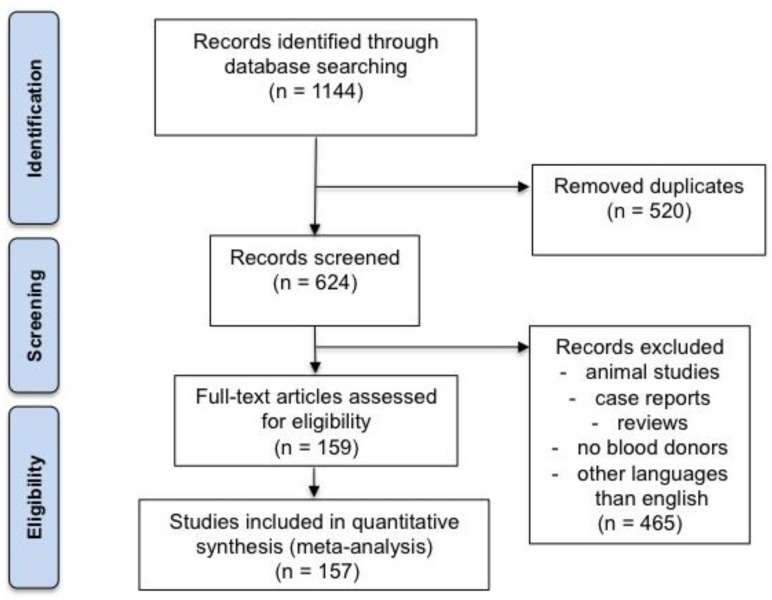
Study flow chart.

Out of 1144 articles, 157 (14%) fulfilled the quality standards and were used in the final analysis, as shown in the flow chart (Figure 1). Detailed information on the studies included and their characteristics are provided as supplementary tables (Supplementary Tables S1 and S2). 

### 3.1. Overall HEV RNA Positivity Rates in Blood Donors

**Figure 2:**
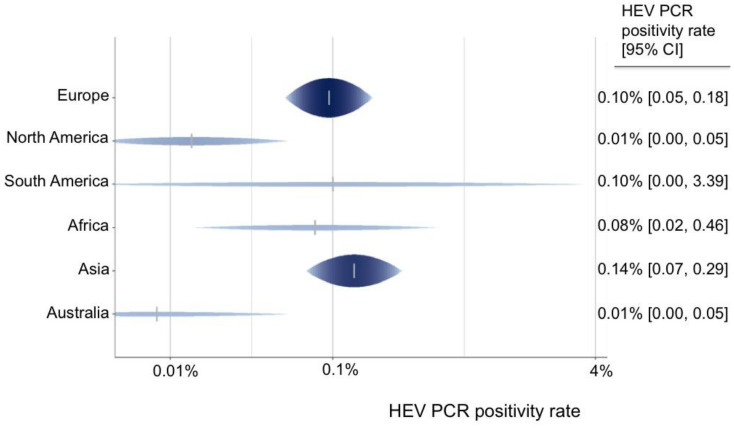
Predicted HEV PCR positivity rate for all continents.

**Figure 3:**
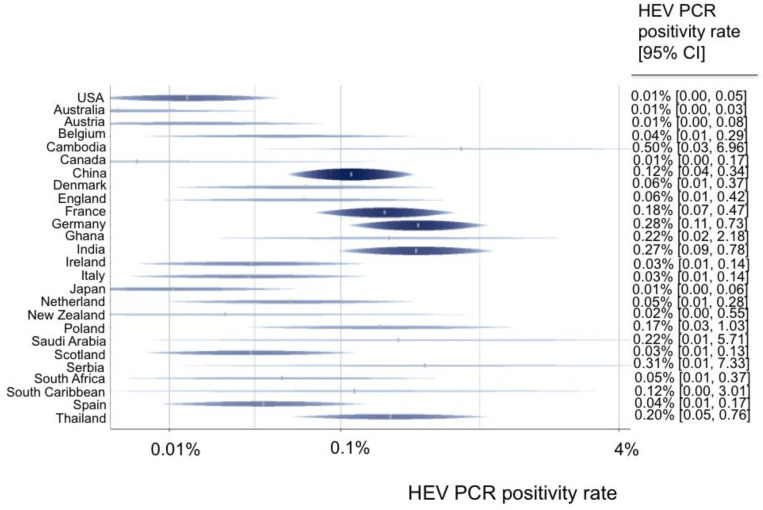
Predicted HEV PCR positivity rates for all countries.

A total of 55 data sets from 44 studies reported the rate of viremia in 3,375,573 blood donors. The HEV RNA positivity varied between continents, and the rate ranged from 0.01% in Australia and North America to 0.14% in Asia (Figure 2). North America (0.01%) had a lower HEV PCR positivity rate in comparison with Europe (0.10%) (OR = 0.14 (95% CI 0.03–0.58), *p*-value = 0.007). Furthermore, the HEV RNA positivity rate varied greatly between single nations, ranging from 0.01% in Australia, the USA, Canada, Austria, and Japan to up to 0.5% in Cambodia, 0.31% in Serbia, and 0.28% in Germany (Figure 3).

Regarding the year of the study, the rate of HEV PCR positivity increased over time from the years 1994 to 2020 (OR = 0.87 (95% CI 0.79–0.96, *p*-value = 0.007)). However, a detailed examination of these results showed that this was only due to data from Europe and did not apply worldwide. The distribution of gender was described in 6 of 44 studies. Adjusted estimates revealed a significantly lower rate of PCR positivity in female versus male blood donors (OR = 0.37 (95% CI 0.20–0.69), *p*-value = 0.002). 

### 3.2. Overall Anti-HEV IgG and IgM Seroprevalence Rates in Blood Donors

A total of 206 data sets from 125 studies reported the anti-HEV IgG seroprevalence in 225,328 blood donors. Eight anti-HEV IgG assays were used in the various studies. In 56 studies, the Wantai assay was used (44.8%); in 18, DiaPro was used (14.4%); in 11 Abbott, was used; in 11, Mikrogen was used (8.8% each); in 4, MP was used (3.2%); in 1, Adaltis was used; in 1, DSI was used (0.8% each); and in 64 studies, other/inhouse or undefined assays (51.2%) were used. 

In 65 data sets, anti-HEV IgM rates were depicted: 25 used Wantai (56.8%), 8 used DiaPro (18.2%), 4 used Mikrogen (9.1%), 2 used MP (4.5%), 1 used DSI (2.3%), and 12 used other/in-house assays/undefined assays (27.3%). Adjusted estimates for group differences regarding the assays demonstrated that the DiaPro (OR = 0.37 (95% CI 0.20–0.69), *p*-value = 0.002), MP (OR = 0.25 (95% CI 0.11–0.58), *p*-value = 0.001), Mikrogen (OR = 0.38 (95% CI 0.18–0.79), *p*-value = 0.01), and various other assays (OR = 0.48 (95% CI 0.31–0.74), *p*-value = 0.001) showed lower seroprevalence rates in comparison with the Wantai assay. 

**Figure 4:**
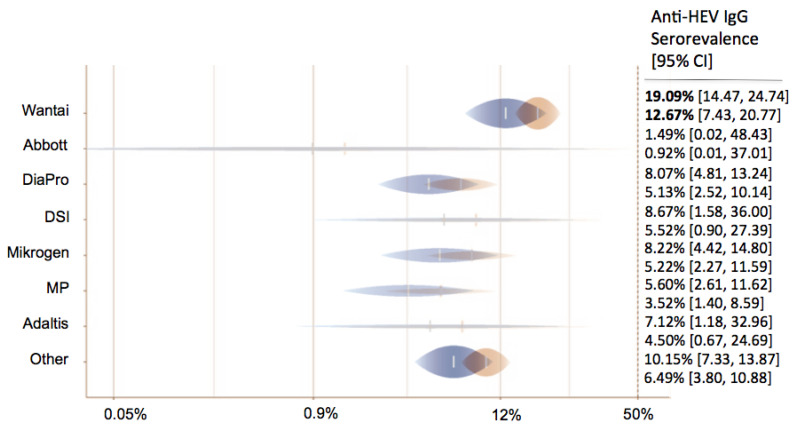
Predicted anti-HEV IgG seroprevalence in North America (blue) and Europe (orange) for all tests.

Depending on the continent, the estimated anti-HEV IgG seroprevalence ranged from 4.79% in Australia to 22.98% in Africa. North America (12.7%, Wantai assay) had a lower IgG seroprevalence in comparison with Europe (19.1%, Wantai assay) (OR = 0.62 (95% CI 0.35–1.09), *p*-value = 0.094) (Figure 4). We did not observe an association between anti-HEV IgG or IgM seroprevalence and the year of the study. The distribution of gender was reported in 45 of 125 studies. In contrast to HEV PCR positivity, there seemed to be no difference regarding anti-HEV IgG or IgM seroprevalence between genders (OR = 0.74 (95% CI 0.53–1.05), *p*-value = 0.089; OR = 0.54 (95% CI 0.21–1.35), *p*-value = 0.186).

## 4. Discussion

Increasing numbers of reported HEV infections, as well as potentially fatal courses in vulnerable patients, have led to the question of whether all blood products should be tested for HEV to avoid blood-borne HEV transmission. While many European countries have already established general blood donor screening for HEV, this has not yet been decided in the U.S. and many other nations; thus, this meta-analysis could help decision-makers worldwide to choose wisely. This large meta-analysis elucidates the rate of HEV PCR positivity and the anti-HEV seroprevalence in blood donors worldwide for the first time.

The highest HEV PCR positivity rates were found in Asia and Europe, and the lowest rates were found in Australia and North America. Furthermore, the HEV viremia rate in South America (0.1%) was slightly lower than that in Europe. However, since this observation was based on only a few data, it should not be overemphasized. Further studies on South America are necessary to realistically assess the risk there. A recent meta-analysis based on patient anti-HEV seroprevalence data already suggested that the risk of HEV exposure in North America might be lower than that in Europe [14]. The current study shows, for the first time, that the anti-HEV positivity and viremia rates in blood donors in North America are lower than those in Europe. Possible reasons could be differences in the type of diet, especially with regard to the amount of pork consumption. By focusing on North America vs. Europe, it was clearly shown that in these two HEV genotype 3 regions, there is a completely different endemicity with strongly differing probabilities for HEV exposure (serology results) and blood-borne HEV transmission (PCR results). Because there is a significantly more inhomogeneous distribution of genotypes in Asia, it is more difficult to compare this continent with other continents.

Furthermore, our data regarding single countries reveals a large variability between viremia rates among blood donors in numerous European countries. The highest rates were detected in Serbia, Germany, and France. This observation is in line with that of Hartl et al., who also found the highest anti-HEV seroprevalence in France and Germany in Europe in the general population [2]. Regarding a possible sex difference, male blood donors showed significantly higher HEV PCR positivity rates than female blood donors. However, this analysis was based only on available data from a few studies (*n* = 6) from five different countries (Germany, Ireland, Italy, South Africa, and Thailand). Therefore, this observation needs to be confirmed and should not be overemphasized. On the other hand, these studies include 94,386 female (40%) and male (60%) blood donors, which represent a representative cohort. Sex differences could be related to HEV exposure, such as potentially higher rates of consumption of insufficiently heated pork in men [4,15], hormonal differences affecting the immune system [16] or socioeconomic factors, and the distribution of different genders in different occupational groups. The increasing HEV PCR positivity rate we observed was caused by European countries only. Thus, this finding should not be overestimated and cannot be generalized. Perhaps this observation was caused by the increased sensitivity of assays used and does not depict a real epidemiological shift. 

This work contributes significantly to the current question of whether blood products worldwide should be tested for HEV. According to the viremia rate in Europe (0.10%) in comparison to North America (0.01%), it is apparent that the risk of HEV transmission by blood products is far lower in North America than in Europe. Therefore, it may be speculated that general blood donor screening for HEV is not indicated in the U.S. in contrast to many European countries (Figure 3). Certainly, the characteristics of the European nations are so inhomogeneous (Figure 3; Supplementary Tables S1 and S2) that they impede a uniform recommendation for general testing throughout Europe. However, according to the data in Supplementary Table S2, nations worldwide can estimate their risk of transfusion-related HEV transmission and then decide, on this basis, whether to introduce general blood donor screening, initiate further studies, or refrain from testing for HEV. Of course, the risk–benefit analysis as well as the costs must also be taken into account. Certainly, the need to have HEV-free blood products is greater in an industrialized nation with numerous immunosuppressed blood product recipients than in a developing country. 

A limitation of our study is that the age of the studied individuals was not consistently reported in the studies. Standardized analysis of age-dependent effects and lifetime risk could not be performed adequately due to a lack of data. Furthermore, as ethnicity was available only in a minority of studies, no valid conclusion could be drawn regarding this aspect. However, this is the first examination evaluating the risk of blood-borne HEV transmission (PCR positivity) and the risk of HEV exposure (seropositivity) among blood donors worldwide. Both serology and viremia depicted a lower risk of HEV infections in North America in comparison with Europe. The present study meets the high-quality standards set for meta-analyses. All findings were dependent on the quality of the included studies. To avoid potential bias, all data sets were assessed by experienced scientists according to the Joanna Briggs Institute’s critical appraisal tool, which is well-proven for prevalence studies [17].

## Data Availability

Data are available upon reasonable request.

## Supplementary Materials

The following supporting information can be downloaded at: https://www.mdpi.com/article/10.3390/pathogens12030425/s1, Table S1. Studies of HEV PCR positivity rates of blood donors worldwide [18,19,20,21,22,23,24,25,26,27,28,29,30,31,32,33,34,35,36,37,38,39,40,41,42,43,44,45,46,47,48,49,50,51,52,53,54,55,56,57,58,59,60,61]; Table S2. Studies of HEV seroprevalence of blood donors worldwide [62,63,64,65,66,67,68,69,70,71,72,73,74,75,76,77,78,79,80,81,82,83,84,85,86,87,88,89,90,91,92,93,94,95,96,97,98,99,100,101,102,103,104,105,106,107,108,109,110,111,112,113,114,115,116,117,118,119,120,121,122,123,124,125,126,127,128,129,130,131,132,133,134,135,136,137,138,139,140,141,142,143,144,145,146,147,148,149,150,151,152,153,154,155,156,157,158,159,160,161,162,163,164,165,166,167,168,169,170,171,172,173].

## Abbreviations

Hepatitis E virus (HEV), ribonucleic acid (RNA), Preferred Reporting Items for Systematic Reviews and Meta-Analyses (PRISMA), polymerase chain reaction (PCR), odds ratio (OR), confidence interval (CI).
