# Supplementary material for: Higher Risk of HEV Transmission and Exposure among Blood Donors in Europe and Asia in Comparison to North America: A Meta-Analysis
Source: Pathogens. 2023 Mar 8;12(3):425. doi: 10.3390/pathogens12030425 (PMC10059948; doi:10.3390/pathogens12030425)
Supplement: Supplementary file 1 [file pathogens-12-00425-s001.zip › pathogens-2249892-supplementary.pdf]

Supplementary

Table S1. Studies of HEV PCR positivity rates of blood donors worldwide.

Author	Year	Country	Number of tested blood donors
Fu et al.[18]	2021	China	1864
Mishra et al.[19]	2021	India	13050
Al Dossary et al.[20]	2021	Saudi Arabia	806
Cordes et al.[21]	2021	Germany	16236
Spreafico et al.[22]	2020	Italy	9726
Maponga et al.[23]	2020	South Africa	10000
Spada et al.[24]	2019	Italy	3912
Tsoi et al.[25]	2019	China	8000
Vercouter et al.[26]	2019	Belgium	38137
Lhomme et al.[27]	2019	France	188082
Harvala et al.[28]	2019	England	1838747
Vollmer et al.[29]	2019	Germany	7650
Intharasongkroh et al.[30]	2019	Thailand	30112
Katiyar et al.[31]	2018	India	1799
Wen et al.[32]	2018	China	5345
Spada et al.[33]	2018	Italy	9141
Thom et al.[34]	2018	Scotland	94302
Westhölter et al.[35]	2018	Germany	18737
Grabarczyk et al.[36]	2018	Poland	12664
Hewitt et al.[37]	2018	New Zealand	5000
Hoad et al.[38]	2017	Australia	74131
Roth et al.[39]	2017	USA	128020
Fearon et al.[40]	2017	Canada	13993
Gallian et al.[41]	2017	France	31879
Minagi et al.[42]	2016	Japan	512564
Shrestha et al.[43]	2016	Australia	14799
Vollmer et al.[44]	2016	Germany	16125
O´Riordan et al.[45]	2016	Ireland	24985
Nouhin et al.[46]	2016	Cambodia	301
Harritshøj et al.[47]	2016	Denmark	25637
Schreuder et al.[48]	2015	South Caribbean	600
Stramer et al.[49]	2015	USA	18829
Fischer et al.[50]	2015	Austria	58915

Sauleda et al.[51]	2015	Spain	9998
Petrović et al.[52]	2014	Serbia	200
Slot et al.[53]	2013	Netherland	40176
Xu et al.[54]	2013	USA	1939
Cleland et al.[55]	2013	Scotland	43560
Meldal et al.[56]	2013	Ghana	239
Vollmer et al.[57]	2012	Germany	200
Guo et al.[58]	2010	China	44816
Herremans et al.[59]	2007	Netherland	50
Khuroo et al.[60]	2004	India	107
Arankalle et al.[61]	1999	India	200

Table S2. Studies of HEV seroprevalence of blood donors worldwide.

Author	Year	Country	Number of tested blood donors
Al Dossary et al.[20]	2021	Saudi Arabia	806
Costa et al.†[62]	2021	Brazil	80
Fu et al.†[18]	2021	China	1864
Wong et al.†[63]	2021	Malaysia	1003
Baymakova et al.[64]	2021	Bulgaria	555
Di Lello et al.[65]	2020	Argentina	391
Maponga et al.[23]	2020	South Africa	250
Bangueses et al.[66]	2020	Uruguay	400
Spreafico et al.[22]	2020	Italy	767
Capai et al.[67]	2020	France	2705
Spada et al.†[24]	2019	Italy	3912
Yrondi et al.†[68]	2019	France	99
Arce et al.[69]	2019	Argentina	813
Capai et al.[70]	2019	France	467
Jupattanasin et al.[71]	2019	Thailand	630
Yaşar et al.[72]	2019	Turkey	4022
Moss da Silva et al.[73]	2019	Brazil	281
Miletić et al.†[74]	2019	Croatia	1036
Twagirumugabe et al.[75]	2019	Rwanda	642
Slavov et al.[76]	2019	Brazil	91
Chen et al.†[77]	2019	China	4041
Dimeglio et al.†[78]	2018	French Polynesia	300
Tripathy et al.†[79]	2018	India	2447
Niederhauser et al.[80]	2018	Switzerland	3609
Katiyar et al.[31]	2018	India	613

Hardtke et al.[81]	2018	Brazil	199
Al-Absi et al.†[82]	2018	Qatar	1049
Bura et al.[83]	2018	Poland	110
Wen et al.†[32]	2018	China	5345
Spada et al.[33]	2018	Italy	9141
Mooij et al.[84]	2018	Netherland	1562
Thom et al.[34]	2018	Scotland	1714
Grabarczyk et al.†[36]	2018	Poland	3079
Hewitt et al.[37]	2018	New Zealand	1013
Juhl et al.[85]	2017	Germany	357
Galli et al.[86]	2017	Italy	1370
Bura et al.[87]	2017	Poland	105
Passos-Castilho et al.[88]	2017	Brazil	500
Bura et al.[89]	2017	Poland	246
Pandolfi et al.[90]	2017	Brazil	780
Gupta et al.†[91]	2016	Nepal	581
Nasrallah et al.†[92]	2017	Qatar	5854
Slot et al.[93]	2017	Netherland	850
Fearon et al.[40]	2017	Canada	4102
Lopes et al.†[94]	2017	South Africa	300
Wang et al.†[95]	2017	China	4046
Abravanel et al.†[96]	2017	France	600
De Sabato et al.[97]	2017	Italy	170
Shrestha et al.†[98]	2016	Nepal	1845
Lange et al.†[99]	2016	Norway	1200
Parsa et al.†[100]	2016	Iran	700
O´Riordan et al.[45]	2016	Ireland	1071
Lucarelli et al.[101]	2016	Italy	313
Nouhin et al.†[46]	2016	Cambodia	301
Heamizadeh et al.[102]	2016	Iran	559
Mansuy et al.†[103]	2016	France	10569
Traoré et al.†[104]	2016	Burkina Faso	1497
Ricco et al.†[105]	2016	Italy	199
Naeimi et al.[106]	2015	Iran	628
Schreuder et al.†[48]	2015	South Caribbean	600
Norder et al.†[107]	2015	Sweden	500
Puttini et al.[108]	2015	Italy	132
Sarkar et al.[109]	2015	USA	63

Passos-Castilho et al.[110]	2015	Brazil	300
Mansuy et al.†[111]	2015	France	3353
Holm et al.[112]	2015	Denmark	504
Fischer et al.[50]	2015	Austria	1203
Sauleda et al.[51]	2014	Spain	1082
Shrestha et al.[113]	2014	Australia	3237
Ben-Ayed et al.[114]	2014	Tunisia	426
Petrović et al.[52]	2014	Serbia	158
Zhuang et al.[115]	2014	China	486
Hogema et al.[116]	2014	Netherland	6488
Pittaras et al.[117]	2014	Greece	265
Ren et al.†[118]	2014	China	9719
Jahromi et al.†[119]	2013	Iran	477
Ramezani et al.†[120]	2013	Iran	52
Slot et al.[53]	2013	Netherland	5239
Johargy et al.†[121]	2013	Saudi Arabia	900
Xu et al.†[54]	2013	USA	1939
Ehteram et al.[122]	2013	Iran	530
Cleland et al.†[55]	2013	Scotland	2087
Scotto et al.†[123]	2013	Italy	151
Juhl et al.[124]	2013	Germany	1019
Traoré et al.[125]	2012	Burkina Faso	178
Meldal et al.†[56]	2012	Ghana	239
Vollmer et al.†[57]	2012	Germany	200
Silva et al.[126]	2012	Brazil	110
Cheng et al.[127]	2012	China	546
Neffati et al.[128]	2012	Tunisia	687
Dremsek et al.[129]	2011	Germany	298
Mansuy et al.[130]	2011	France	512
Dong et al.[131]	2011	USA	372
Krumbholz et al.[132]	2011	Germany	116
Kaufmann et al.[133]	2011	Switzerland	550
Beale et al.[134]	2010	England	262
Takeda et al.[135]	2010	Japan	12600
Guo et al.†[58]	2010	China	44816
Masia et al.[136]	2009	Italy	402
Christensen et al.[137]	2008	Denmark	630
Dalton et al.[138]	2008	England	500

Mansuy et al.[139]	2008	France	529
Assarehzadegan et al.[140]	2008	Iran	400
Taremi et al.[141]	2007	Iran	399
Dalton et al.[142]	2007	New Zealand	265
Boutrouille et al.[143]	2007	France	1998
Herremans et al.†[59]	2007	Netherland	50
Bortoliero et al.[144]	2006	Brazil	996
Fukuda et al.[145]	2004	Japan	4256
Khuroo et al.[60]	2004	India	107
Engle et al.[146]	2002	USA	230
Meng et al.[147]	2002	USA	400
Kiesslich et al.[148]	2002	Brazil	227
Trinta et al.[149]	2001	Brazil	93
Arankalle et al.†[61]	2000	India	412
Gonçales et al.[150]	2000	Brazil	165
Lemos et al.†[151]	2000	Cuba	1149
Jutavijittum et al.[152]	2000	Thailand	636
Arankalle et al.[61]	1999	India	200
Karetnyi et al.[153]	1999	USA	443
Konomi et al.†[154]	1999	Bolivia	574
Seow et al.[155]	1999	Malaysia	100
Mateos et al.[156]	1999	Spain	863
Dalekos et al.[157]	1998	Greece	3016
Abdelaal et al.[158]	1998	Saudi Arabia	593
Pavia et al.[159]	1998	Italy	360
Araujo et al.[160]	1997	Portugal	341
Mast et al.[161]	1997	USA	5000
Thomas et al.[162]	1997	USA	811
Rey et al.[163]	1997	Argentina	2157
Poovorawan et al.[164]	1996	Thailand	178
Bernal et al.[165]	1995	Spain	492
Zaaijier et al.[166]	1995	Netherland	1275
Moavenm et al.[167]	1995	Australia	279
Peng et al.[168]	1995	Taiwan	281
Zanetti et al.[169]	1994	Italy	948
Lavanchy et al.[170]	1994	Switzerland	94
Gajjar et al.‡[171]	2014	India	460

Utba et al.‡[172]	2013	Iraq	212
Ibrahim et al.‡[173]	2011	Egypt	760

† anti-HEV IgG and IgM

‡ only anti-HEV IgM
